# Empathy Toward Artificial Intelligence Versus Human Experiences and the Role of Transparency in Mental Health and Social Support Chatbot Design: Comparative Study

**DOI:** 10.2196/62679

**Published:** 2024-09-25

**Authors:** Jocelyn Shen, Daniella DiPaola, Safinah Ali, Maarten Sap, Hae Won Park, Cynthia Breazeal

**Affiliations:** 1 MIT Media Lab Cambridge, MA United States; 2 Language Technologies Institute School of Computer Science Carnegie Mellon University Pittsburgh, PA United States

**Keywords:** empathy, large language models, ethics, transparency, crowdsourcing, human-computer interaction

## Abstract

**Background:**

Empathy is a driving force in our connection to others, our mental well-being, and resilience to challenges. With the rise of generative artificial intelligence (AI) systems, mental health chatbots, and AI social support companions, it is important to understand how empathy unfolds toward stories from human versus AI narrators and how transparency plays a role in user emotions.

**Objective:**

We aim to understand how empathy shifts across human-written versus AI-written stories, and how these findings inform ethical implications and human-centered design of using mental health chatbots as objects of empathy.

**Methods:**

We conducted crowd-sourced studies with 985 participants who each wrote a personal story and then rated empathy toward 2 retrieved stories, where one was written by a language model, and another was written by a human. Our studies varied disclosing whether a story was written by a human or an AI system to see how transparent author information affects empathy toward the narrator. We conducted mixed methods analyses: through statistical tests, we compared user’s self-reported state empathy toward the stories across different conditions. In addition, we qualitatively coded open-ended feedback about reactions to the stories to understand how and why transparency affects empathy toward human versus AI storytellers.

**Results:**

We found that participants significantly empathized with human-written over AI-written stories in almost all conditions, regardless of whether they are aware (t_196_=7.07, *P*<.001, Cohen *d*=0.60) or not aware (t_298_=3.46, *P*<.001, Cohen *d*=0.24) that an AI system wrote the story. We also found that participants reported greater willingness to empathize with AI-written stories when there was transparency about the story author (t_494_=–5.49, *P*<.001, Cohen *d*=0.36).

**Conclusions:**

Our work sheds light on how empathy toward AI or human narrators is tied to the way the text is presented, thus informing ethical considerations of empathetic artificial social support or mental health chatbots.

## Introduction

Empathy, the sharing of emotions with a social other, is foundational in developing strong interpersonal ties [1,2] and mental well-being [3]. With the rise of large language models (LLMs) and increase in chatbots for social companionship [4] and mental health [5,6], it is crucial to understand how empathy toward artificially intelligent agents manifests and what the social implications of this phenomenon are. In particular, commercial chatbots often display anthropomorphism by adopting their own identities or experiences. Current artificial intelligence (AI) systems hold the ability to express social and emotional influences through the mechanisms of empathy, which can lead to downstream impacts in the real world. For example, in AI service applications, increased empathy improves service acceptance and user compliance [7,8]. Such empathetic relationships with synthetic agents can unfold in the following ways: (1) in the behavior of the agent, where the agent behaves in an empathic way toward other agents and toward the user, and (2) in the relation the agent establishes with the user, where the agent looks like and acts in a way that leads the user to establish an empathic relation with it [9,10]. Prior works indicate that acceptance and trust toward AI devices is directly related to how much people empathize with an AI system [9]. Further works validate that the more personal information AI agents disclose, the more empathetic human conversation partners are toward these agents [11]. As machines are increasingly capable of telling human-like stories in daily life, this raises important questions about how people might empathize with AI-written stories in real-world, user-facing contexts, comparing them with how people perceive stories created by AI, and how transparency of the system modulates these psychological responses [12,13].

In this work, we study how much people empathize with stories created by AI compared with stories created by other humans as well as how author disclosure affects perceived empathy. Humans can breathe life into inanimate or artificial systems [9,14-16] and are able to relate to fictional experiences when they are human-like or realistic in the scope of one’s own life [17,18]. As such, this work calls for ethical and philosophical concerns about differences in empathy toward humans and AI—machines have no lived experiences yet can produce stories as their “own” [19,20]. If the machine uses these fabricated experiences to elicit a particular behavior from the user, is this considered manipulation? How are behaviors shifted if the user is aware that the experiences are fabricated? How might empathy toward AI agents, such as social companion or mental health chatbots, impact downstream human outcomes?

Because outputs from generative AI are not an artificial agent’s actual experiences [20], but rather a probabilistically sampled sequence of text from human experiences, it is important to be precise and nuanced when communicating results from generated text to ensure ethical deployment of such systems in the mental health domain [21-23]. In the field of social psychology, researchers have explored how nudges, small subtle changes that can inspire big changes in actions, can modulate empathy [24]. Yet, in the AI domain, few works have explored how subtle design changes in the presentation of AI-written stories can significantly shift attitudes and empathy toward AI systems [25].

Prior works generally indicate that perceptions of AI can change depending on transparency. Most works find that knowledge of AI involvement reduces the perception of the agent or quality of interaction and that there are fundamental qualities of “humanness” in texts written by people [25-27], but that fostering trust and acceptance can lead to more empathy toward an AI agent [9]. Grounded by these works, we hypothesize that empathy toward AI-written stories, both generated and retrieved in response to a user’s own personal story, will be significantly lower than empathy toward human-written stories whether the author is disclosed [H1]. We also hypothesize that people will be more willing to empathize with AI stories when the author of the story is made transparent, as the output could be perceived as more trustworthy [H2]. To test our hypotheses, in this work, we investigate the following:

1. How does empathy change when stories, human- or AI-written, are retrieved versus generated directly by a language model?
2. How does transparency about the author of a story play a role in empathy toward human versus AI narrators?

In summary, we aim to answer the following research questions:

- How does empathy toward human- versus AI-written stories differ?
- What qualities of human- versus AI-written stories affect people’s empathetic responses?
- How do the aforementioned results change when the narrator of a story is made transparent to users?
- What are the ethical implications of empathy toward AI stories in social support and mental health chatbots, and how are these implications influenced by transparency of the story’s author?

## Methods

### Study Procedure

We conducted 4 crowd-sourced studies with a total of 985 participants to assess the effects of author origin on empathy. Within each session, participants wrote their own personal stories and rated empathy toward stories written by people or by ChatGPT.

The retrieved stories were matched based on similarity of the embeddings of stories, and generated stories were generated on the fly, given the user’s story as a prompt. We used ChatGPT to generate a set of stories using seed stories from the EmpathicStories data set [28]. Stories generated by ChatGPT (gpt-3.5-turbo) were prompted with a context story and the following instruction: Write a story from your own life that the narrator would empathize with. Do not refer to the narrator explicitly.

The study’s 4 comparisons are as follows:

- H-CR: we compared empathy toward the narrator across *human-written retrieved* stories and *ChatGPT-retrieved* stories
- H-CR+T: we compared empathy toward the narrator across *human-written retrieved* stories and *ChatGPT-retrieved* stories, making transparent to the user whether the story they read was written by a human or AI before they rated their empathy (repeat H-CR with transparency)
- H-CG: we compared empathy toward the narrator across *human-written retrieved* stories and *ChatGPT-generated* stories (in response to the user’s story as a context).
- H-CG+T: we compared empathy toward the narrator across *human-written retrieved* stories and *ChatGPT-generated* stories, making transparent to the user whether the story they read was written by a human or AI before they rated their empathy (repeat H-CG with transparency).

Finally, in all studies, participants reported how their empathy toward the stories would change if the stories were written by AI. We define human-written retrieved stories as personal stories that are written by people and retrieved using a model that takes the user’s self-written story as a query. ChatGPT-retrieved stories come from our pregenerated set of 1568 stories and are retrieved in the same manner as human-written retrieved stories. Finally, ChatGPT-generated stories are stories that are generated on the fly, given the user’s story as input. Examples of stories across conditions are shown in Textbox 1.

**Examples of a user story (written by a participant) and the corresponding retrieved human-written story, pregenerated ChatGPT story retrieved based on the user’s story, and on-the-fly generated ChatGPT story.**

**User story**

“I started to experience a nagging pain in my right upper abdomen...The GP sent me for an ultrasound which detected hydronephrosis and referred me to a consultant...Following more scans I was found to have multiple spinal cysts in the whole spine...I felt so grateful and validated that someone had finally helped me...I am starting to finally feel at peace that I have a reason and I am not crazy.”

**Human-written retrieved story**

“A week ago, I was having pain in my back. I thought it was just normal wear and tear until the pain got worse...When I was waiting for the doctor, a nurse came in the room...She said that I had kidney stones...I was so relieved after that. I felt like I had a new lease on life, and decided to commit myself to eating healthier and drinking much more water.”

**ChatGPT-written retrieved story**

“I remember the first time I felt a strange pain in my chest...At first, I thought it might be a heart attack, but I was young and healthy,...Finally, after months of searching, I found a specialist who was able to give me some answers...It turned out that I had a rare condition...It was a relief to finally have a diagnosis, but it was also a shock to realize that my instincts had been right all along.”

**ChatGPT-written story**

“I remember the first time I felt a strange pain in my chest...At first, I thought it might be a heart attack, but I was young and healthy,...Finally, after months of searching, I found a specialist who was able to give me some answers...It turned out that I had a rare condition...It was a relief to finally have a diagnosis, but it was also a shock to realize that my instincts had been right all along.”

### Ethical Considerations

Our study was approved by the Massachusetts Institute of Technology’s ethics board as an exempt protocol (ID E-4618).

### System and Interaction Design

#### Story Prompts and Retrieved Stories

To prompt vulnerable and meaningful personal stories from users, we used questions from the Life Story Interview, an approach from social science that gathers key moments from a person’s life [29] (eg, “Look back over your life and tell us and emotional moment or experience you had in the past...”). In order to ensure that topics were constrained to stories present in our retrieval database, we used topic modeling to identify key clusters in the personal narratives from EmpathicStories. To identify these topics, we used Latent Dirichlet Allocation and KeyBERT on the clusters [30]. Users were instructed to reflect on their life in relation to one of the chosen topics (eg, family and mental health). Personal stories that participants wrote were restricted to be between 500 and 10,000 characters. See Multimedia Appendix 1 for full participant instructions. Stories retrieved by our model were either pulled from the EmpathicStories data set (1568 stories) or generated by ChatGPT (gpt-3.5-turbo). These stories covered a diverse set of personal experiences including mental health, life changes, loneliness, depression, substance abuse, and trauma.

#### Story Retrieval Model

Because our study aimed to assess differences in empathy toward human- versus AI-written stories, both the user’s experiences and the stories returned by our system were important. Returning a story at random could undermine the user’s experiences and hinder their empathy toward the retrieved story. Although many methods exist to retrieve semantically similar pieces of text [31], few focus on retrieving stories that users would emotionally resonate with, given their own story context. As such, we used a fine-tuned BART-base model from Shen et al [28], which was trained on the EmpathicStories data set, a corpus containing pairs of stories each annotated with an “empathic similarity” score from 1 to 4, where empathic similarity refers to how likely the narrators of both stories would empathize with one another. Using this model, we improved retrieval of stories that are empathetically relevant to a user’s own personal story.

#### User Study Interface

We deployed a web interface similar to a guided journaling application where users write and read personal stories. The interface connects to a server run on a graphics processing unit machine (4x Nvidia A40s, 256 GB of RAM, and 64 cores; Nvidia), which retrieves story responses in real time. In addition, the server connects the front end to Firebase Realtime storage in order to track interaction data throughout the course of the study.

### Participants and Recruitment

We recruited a pool of 985 participants from Prolific, a crowdsourcing platform that connects researchers with survey respondents for high-quality data collection. Participants across the studies were predominantly female and White. All participants on average had high trait empathy and neutral arousal and valence before starting the study. Full demographic distributions across the 4 studies are shown in Table 1. All participants were paid US $12 per hour for their time.

**Table 1:** Participant demographic distribution.

Demographic			H-CG		H-CR		H-CR+T		H-CG+T
Number of participants			300		299		197		189
Age (y), mean (SD), range			37.60 (12.54), 18-75		40.18 (14.31), 18-79		40.16 (13.76), 19-77		38.82 (13.52), 18-79
**Gender, n (%)**									
	Women	173 (58)		161 (54)		100 (51)		111 (58)	
	Men	120 (40)		132 (44)		93 (47)		76 (40)	
	Nonbinary	5 (1)		3 (1)		2 (1)		1 (1)	
	N/A^a^	2 (1)		3 (1)		2 (1)		1 (1)	
**Ethnicity, n (%)**									
	White	228 (76)		242 (81)		160 (81)		145 (77)	
	Black	24 (8)		16 (5)		20 (10)		13 (7)	
	Asian	14 (5)		15 (5)		6 (3)		9 (5)	
	Other	13 (4)		7 (2)		4 (2)		5 (2)	
	Indian	10 (3)		7 (2)		4 (2)		13 (7)	
	NA	5 (2)		8 (3)		3 (2)		1 (0.05)	
	Hispanic	4 (1)		2 (1)		—^b^		1 (0.05)	
	Middle Eastern	1 (0.05)		1 (0.05)		—		2 (1)	
	Native	1 (0.05)		—		—		—	
	Islander	—		1 (0.05)		—		—	
Empathy level, mean (SD), range			4.26 (0.83), 1-5		4.18 (0.79), 1-5		4.31 (0.79), 1-5		4.24 (0.69), 2-5
Arousal, mean (SD), range			4.42 (1.84), 1-9		4.80 (1.78), 1-9		4.81 (1.94), 1-9		4.48 (1.94), 1-9
Valence, mean (SD), range			5.75 (1.68), 1-9		5.76 (1.70), 1-9		5.75 (1.86), 1-9		5.76 (1.58), 1-9

^a^N/A: not applicable.

^b^—: not available.

### Data Collection and Analysis

**Figure 1:**
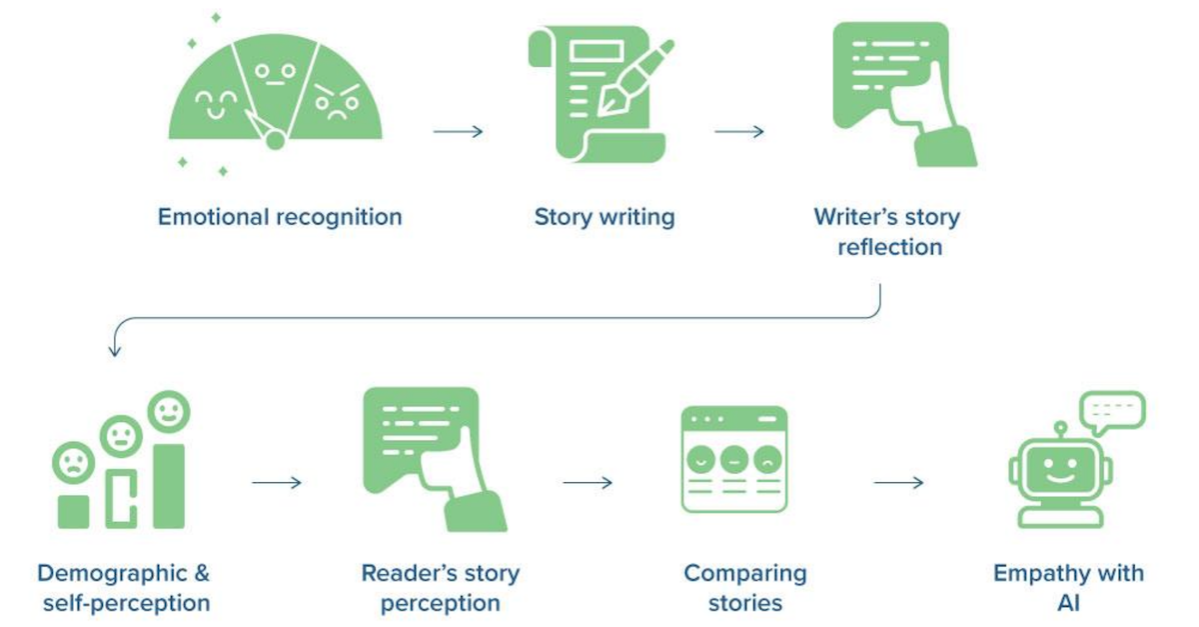
Flow of the user study procedure. Participants recognize their own emotions, write their personal stories, reflect on the stories, and then fill out demographic and self-perception of state empathy information in a survey. Then, they read and perceive given stories, compare and rank the stories, and rate how willing they are to empathize with artificial intelligence (AI) in general.

At the beginning of the study, we measured the user’s valence and arousal using a self-report 9-point Likert scale, as current emotional state could influence empathy. Participants on average had neutral arousal and valence, so we did not exclude any participants from the study. For our empathy measurement, we used a shortened version of the State Empathy Scale [32], which contains 7 questions covering affective (sharing of others’ feelings), cognitive (adopting another’s point of view), and associative (identification with others) aspects of situational empathy. Users additionally provided free-text responses about their empathy toward the story as well as multiple-choice questions listing reasons why they did or did not empathize with the story (ie, the feelings the narrator had, the authenticity of the story, or providing free response reasons). At the end of the study, users self-reported how their empathy would change if the stories they read in the session were written by AI (which we term as perceived empathy with AI). Finally, at the end of the study, we collected demographic information including trait empathy, age, gender, and ethnicity. All survey questions were mandatory for participants (with “prefer not to answer” options for sensitive demographic information). Experiments were conducted throughout the month of June 2023. A full flow of the user study procedure is shown in Figure 1.

We used both quantitative and qualitative approaches to understand the effects of empathy toward a story from human versus AI narrators and offer insights around why empathy shifts under certain conditions. To analyze differences in empathy with the State Empathy Scale, we used a 2-tailed paired *t* test, as we identified through a Shapiro-Wilke test that the data are normally distributed. Note that we computed total empathy toward a story using the mean of the State Empathy Scale survey questions, and we present descriptive analyses of survey responses across conditions as mean (SD). To compare perceived empathy across studies, we used an independent, 2-tailed *t* test. All quantitative analyses were conducted via statistical libraries written in Python (SciPy Statistics). For qualitative analysis, open-ended explanations for the empathy rating were thematically coded using an inductive approach [33]. Two researchers (JS and DP) independently coded a subset of the data using Excel spreadsheets (Microsoft Corp) and reached substantial agreement with a Cohen κ value of 0.70.

## Results

### Effects of Human Versus AI Authorship on Empathy Toward Stories

#### Participants Generally Felt More Empathy for Human-Written Stories Than AI-Written Stories

When we instead retrieved stories from a corpus of narratives generated by ChatGPT (H-CR), total empathy decreased across AI-written (mean 3.83, SD 0.73) versus human-written (mean 4.01, SD 0.72) stories (t_298_=3.46, *P*<.001, Cohen *d*=0.24). This indicates a noticeable difference between human- versus. AI-written stories, which we explore further through qualitative analysis in later sections. When we made transparent to the user the author of the retrieved story (H-CR+T), we saw an even greater decrease in total empathy toward AI-written (mean 3.61, SD 0.85) stories relative to human-written (mean 4.1, SD 0.69) stories (t_196_=7.07, *P*<.001, Cohen *d*=0.60).

When comparing *human-retrieved* (mean 3.96, SD 0.78) stories and *ChatGPT-generated* stories (mean 4.30, SD 0.65) based on the user’s original story (H-CG), we found that participants empathized significantly more with ChatGPT-generated stories than human-retrieved stories (t_299_=6.14, *P*<.001, Cohen *d*=0.47). Following this trend, we found that there was no statistically significant difference between empathy toward *human-retrieved* (mean 3.93, SD 0.73) and *ChatGPT-generated* (mean 4.02, SD 0.71) stories when the author was made transparent (H-CG+T).

#### Generated Stories Elicit More Empathy Than Retrieved Stories

**Figure 2:**
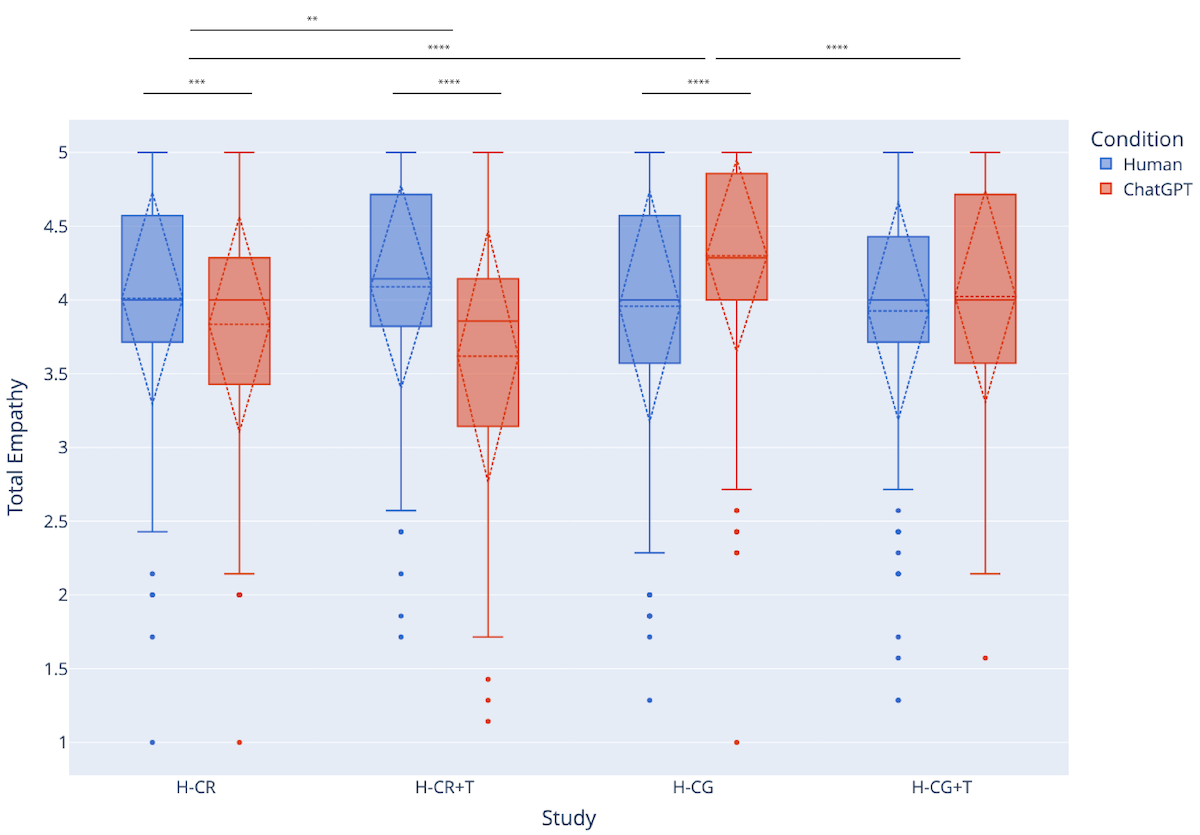
Changes in total empathy toward stories participants read across conditions (human-written vs AI-written story) and studies (author made transparent vs author not transparent, AI story was retrieved vs generated). *P<.05, **P<.01, ***P<.001, ****P<.000.

Next, we cross-compared total empathy toward AI-written stories in H-CG (mean 4.3, SD 0.65) and H-CR (mean 3.83, SD 0.73), allowing us to explore differences in ChatGPT responding directly to a user’s personal story context as compared with retrieving a relevant AI-generated story. From Figure 2, we see that empathy statistically significantly decreases in H-CR when stories are retrieved instead of generated directly from the user’s written story (t_597_=8.20, *P*<.001, Cohen *d*=0.67).

#### Disclosure of Story Author Reduces Empathy in ChatGPT-Generated Stories

We cross-compared total empathy toward AI-written stories in H-CR and H-CR+T (mean 3.62, SD 0.86), allowing us to assess how transparency about a story being written by ChatGPT shifts empathy. We found that empathy toward the AI-written stories statistically significantly decreased when users were told before reading that the story was written by ChatGPT (t_494_=3.02, *P*<.001, Cohen *d*=0.27), as shown in Figure 2.

Finally, we cross-compared total empathy toward AI-written stories in H-CG and H-CG+T (mean 4.02, SD 0.72) and saw that empathy in H-CG+T statistically significant decreased (t_487_=4.37, *P*<.001, Cohen *d*=0.40). This confirmed the aforementioned result that telling participants a story was written by AI decreases empathy (Figure 2).

### Effects of Author Disclosure on Willingness to Empathize With AI: People Are More Willing to Empathize With AI-Written Stories if Author Is Transparent

**Figure 3:**
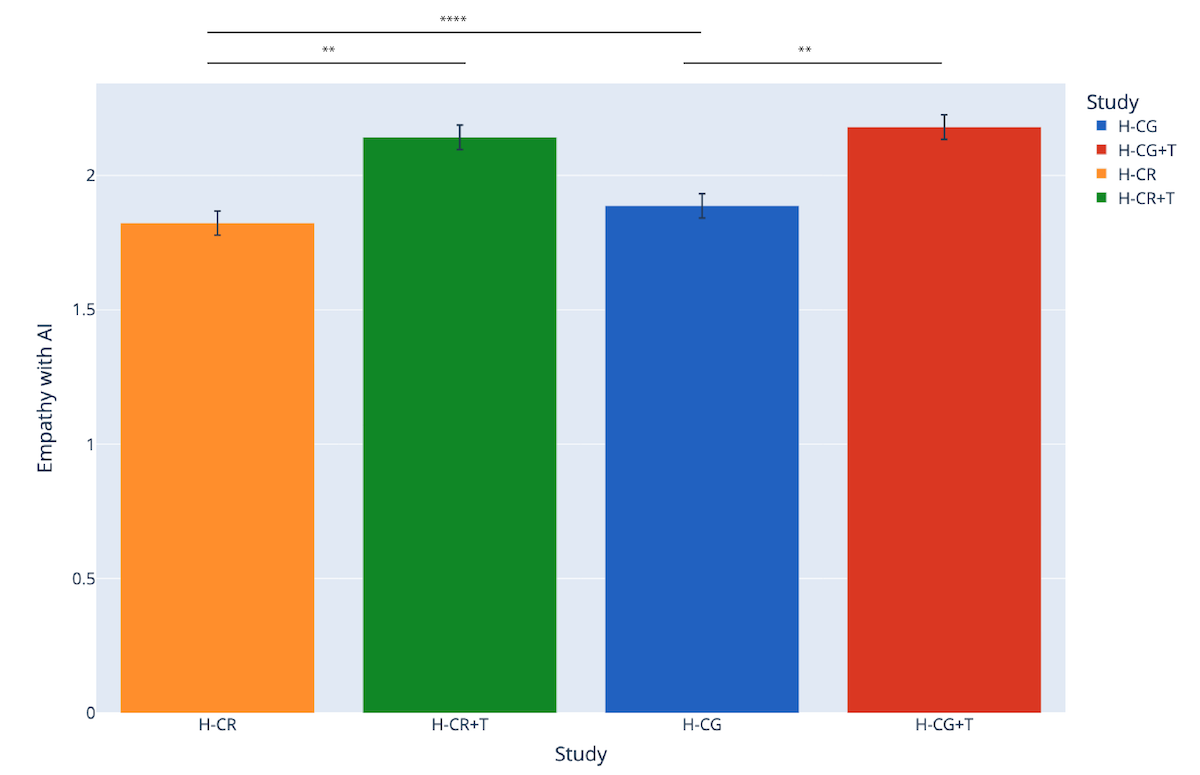
Self-reported willingness to empathize with AI-written stories across all 4 studies. AI: artificial intelligence. *P<.05, **P<.01, ***P<.001, ****P<.000.

In addition to raw, self-reported empathy toward the narrator of each story, we also asked participants to rate how much they believe their empathy would shift if the stories they read were all written by AI, where scores are from Likert 1 (empathize a lot less) to 4 (empathize a lot more). As shown in Figure 3, we find that across all 4 studies, participants would, on average, empathize less (scores are generally at or below 2) with AI-written stories using our survey measurements (H-CG: mean 1.88, SD 0.91; H-CR: mean 1.82, SD 0.90; H-CR+T: mean 2.14, SD 0.89; H-CG+T: mean 2.18, SD 0.87). However, interestingly, we see that willingness to empathize with AI-written stories statistically significantly increases when we are transparent about the story being written by ChatGPT (ie, participants read a story knowing it was generated by ChatGPT). These results are shown in cross-comparing H-CR and H-CR+T for retrieved ChatGPT stories (t_494_=–5.49, *P*<.001, Cohen *d*=0.36) as well as cross-comparing H-CG and H-CG+T for directly generated ChatGPT stories (t_494_=–4.99, *P*<.001, Cohen *d*=0.33).

### Understanding Mechanisms Behind Empathy Toward Human Versus AI Stories

Through qualitative coding of participant free responses, conducted by 2 independent coders, we revealed 9 unique themes around why participants did or did not empathize with the stories (Table 2). Participants explained their reasoning by commenting on the narrator’s perspective, including empathizing with the *situation* in the story or the *emotions* the narrator describes. Some participants expressed that the story was *not relatable* enough for them to empathize with. Two themes appeared around the way that the story was written: some expressed *story confusion* due to some of the logic of the story not being clear, and there was also a common theme around the *word choice* of the narrator, such as the writing style or phrasing. There were some participants who *mentioned AI* explicitly, and others who talked about the *authenticity* of the story, or whether it was real or fake. Some participants spoke about their *personality* being a factor in whether they were able to empathize. The *other* category was used if a response did not fit into an existing category.

We assigned a theme (or themes) to each response, and a percentage was calculated to account for the number of participants in each study. As a whole, the *emotional* (30.38%) and *situational* (24.74%) codes showed up most frequently across all conditions. One notable difference is that participants in H-CG (35.38%) and H-CR (31.99%) had a higher percentage of *emotional* codes than H-CR+*t* (23.40%) and H-CG+*t* (27.29%). H-CR+T and H-CG+T had a higher percentage of *word choice*, *authenticity*, and *mention AI* codes than H-CG and H-CR.

We broke themes down into individual studies and conditions. Conditions H-CR and H-CR+T were compared, as they compared the same types of stories (human-retrieved vs AI-retrieved), with H-CR+T explicitly telling participants when the stories were AI generated. Interestingly, codes for *emotional* were less common in the H-CR+T condition. H-CG and H-CG+T were compared (human-retrieved vs AI-generated) and showed a similar decrease in *emotional* codes.

**Table 2:** Themes resulting from a qualitative analysis across all four studies^a^.

Code	Definition	Example quote	Total (%)	H-CG (%)	H-CR (%)	H-CG+T (%)	H-CR+T (%)
Emotional	Empathize with the emotions that the narrator describes in the story	“I can recognize the fact that someone would feel conflicted in this scenario. Simultaneously happy and envious.”	30.38^b^	35.38^b,c^	31.99^b^	23.40^b^	27.29^b^
Situational	Empathize with the situation or context that the narrator is in	“The theme of sudden and unexpected loss was similar to my experience. I have lost a parent too which makes it easy to empathize. My parent had a long illness but the end was very sudden, similar to the narrators experience.”	24.74	26.88^c^	25.94	23.19	21.18
Story confusion	Mention of specific details in the story that are not clear, including details or logic that does not add up	“I think her level of self doubt was a bit over the top for a position his finances did not depend on.”	11.41	8.91	12.97^c^	11.49	12.88
Not relatable	Mention of not empathizing because the story was not relatable or they did not agree with the narrator	“I don't want to pursue the same goals as the narrator, so our feelings are different.”	11.20	9.33	14.41^c^	10.43	10.04
Word choice	Mention of the writing style, phrasing, or grammar, typically to reduce feelings of empathy	“It was very poorly written and organized - just a bunch of ideas on the page, rather forced and disjointed. The style did not encourage me to believe it.”	7.39	6.55	5.19	9.57	9.83^c^
Authenticity	Mention of the story being “real” or “fake,” any mention of believability or originality	“It seems genuine, although I am aware that it may not be, as it is a common experience.”	6.67	5.15	4.03	10.85^c^	8.73
Mentions AI	Explicitly mentions AI or automation	“It feels like an AI generated response based on keywords from my experience.”	4.15	3.06	1.15	7.87^c^	6.55
Personality	Mention of personal ability to empathize	“I am a person who hardly empathizes and more so the story did not feel consistent to me.”	0.47	0.56	0.29	0.43	0.66^c^
Other	Does not fit into any category, restates the question or generic	“I did empathize with the story.”	3.59	4.18^c^	4.03	2.77	2.84

^a^Percentages are shown as the number of times a code was mentioned out of the total number of participants within each study.

^b^The top code in each column.

^c^The top percent in each row.

## Discussion

### Principal Results

From our work, we show that it is important to be intentional in how one presents outputs from generative AI systems. In summary, we find that empathy is higher for ChatGPT-generated stories than ChatGPT-retrieved stories; total empathy toward the story is generally higher for stories written by humans than AI, but that transparency creates greater willingness to empathize with AI.

#### Generated Versus Retrieved Stories

First, through cross-comparisons between ChatGPT-written retrieved stories (H-CR) and ChatGPT-generated stories (H-CG), we find that empathy is higher for ChatGPT-generated stories rather than ChatGPT-retrieved stories. Interestingly, we find that empathy is higher toward ChatGPT-generated stories than human-written retrieved stories. Thus, we did not validate that humans would empathize more with human-written stories in all conditions [H1]. These results on generated versus retrieved stories highlight the importance of context awareness. Generated stories directly respond to the user’s story, and previous literature shows that a direct response to one’s story increases empathy [34]. Output that is generated from conditioning on the stories can take much more from the input story, thus probably reaching a higher level of similarity, beyond what our retrieval algorithm is based on [35]. These results further suggest that trust in the author of the story, rather than the author’s identity, may play a role in empathy toward personal stories.

#### Transparent Versus Opaque Story Author

In studies H-CR and H-CR+T, we find that people significantly empathize less with retrieved AI-written stories than human-written stories, which is in line with and supports previous research findings [26,27]. We find that empathy decreases most between human-written and AI-retrieved stories in H-CR+T when we are transparent about the author of the story. This indicates that knowing when a story is written by AI alters our empathy toward that story and ability to relate to the narrator, possibly because AI is conveying experiences that are not its “own.”

Interestingly, participants’ willingness to empathize with AI systems significantly increases across both retrieval and generation conditions when the author of the story is made transparent (validating [H2]). Prior works indicate that transparency about AI’s lack of human qualities can reduce perceived similarity [27], but that transparency can increase trust toward AI systems [36]. Our results may indicate that disclosing a story’s author could increase willingness to empathize through trust, or through demonstration that AI stories contain relatable qualities.

This finding also has implications for the design of empathetic AI systems outside of therapy. For instance, for social companions, or AI health care providers, where empathy may be an essential social interaction, designers must consider transparency in the AI system to increase users’ willingness to empathize through trust. In practical tool design, this may take the form of disclosing the content creator in the user interactions, such as in the visual user interface or voices used [37]. There is much argument for using more anthropomorphic representations of AI technologies such as robots to increase trust [38], but in our empathetic storytelling interactions, where there is actual uncertainty about the story author, results may indicate otherwise.

In the H-CR+T condition, participants’ reasoning for not empathizing with AI-written stories was more centered around themes relating to how the story was written, including “story confusion” and “word choice,” similar to research that showed that “linguistic style” was a reported indicator for AI-generated text [39]. For example, one participant stated, “The story and feelings described feel really fake and over the top. It does not feel genuine and has clearly been written by a robot.”

Others mention not being able to empathize with the story because the story did not actually happen, but they are still capable of engaging with it as a made-up story. For example, one participant shared, “Because I know it’s written by AI then I can’t think that it is genuine. However, as a work of fiction I can immerse myself in it and connect with the characters portrayed.” This sentiment opens up the potential for AI-written stories to be contextualized for the user in a way that does not feel like they are being deceived by a fake story.

We see no difference in empathy between retrieved human stories and ChatGPT stories generated in direct response to the user (H-CG+T), indicating that responding directly to a user’s story might overshadow the underlying empathic benefits of human-written stories. In this condition, more participants mentioned the “authenticity” of the story or mentioned AI explicitly as a factor against empathizing with the story they read. Participants tended to focus more on the author of the story instead of the content of the story in their open-ended responses. One participant shared, “The story felt similar to the content of my story, which made me feel like I could empathize with it. But knowing the story was written by an AI makes me feel less connected to the story because I know it’s not real.”

### Ethical Considerations and Implications in Mental Health

From our studies, we show that retrieval of human-written stories can encourage human-human empathy rather than empathy toward AI systems, which has broader implications in the digital mental health domain. Large, pretrained generative models do not truly experience the situations present in stories. As such, mental health or social support chatbots powered by AI represent a population sourced from large quantities of human data, but still fall short of human-written stories in their empathic quality [9,13,25,40]. This appropriation of human experiences could be subverted by using AI to instead retrieve more empathically similar texts between human authors [28], such as in social support group settings via web, or to mediate human-human communications, such as between the patient and therapist [41].

To ensure ethical deployment of chatbots and LLMs more broadly in the mental wellness domain, the field of AI has historically advocated for transparency as an ethical design tenet [42]. The more transparent a system is, the more agency one has in the way they use it. However, we show that in framing interactions with stories, a one-sentence disclosure of the author significantly decreased empathy. This finding might be in tension with systems that rely on empathy for efficacy, such as in persuasive technologies that use bonds with AI to improve mental wellness outcomes [15,43]. The empathy and transparency trade-off might not be mutually exclusive, as transparency can breed trust, which also influences interaction. Our work paves directions research should be conducted to understand long-term effects of transparency on the outcomes of chatbots for mental health.

### Limitations and Future Work

The primary limitation in our study design is that not all participants were exposed to all conditions. Given the number of conditions (varying generation or retrieval and transparent or not transparent author), we opted to mix within-subject comparisons and cross-study comparisons, resulting in a less clean study design. However, given the size of our online study, with around 200 participants per study, our results are still statistically sound. Future work can aim to replicate our findings with different study designs to confirm the psychological insights’ soundness.

In addition, given the nature of crowdsourcing and the demographic pool of participants we surveyed, it is important to ensure that findings are replicated in other diverse populations. Although our studies were roughly balanced by gender, prolific respondents are predominantly White. Future work can assess the impact of identity on empathetic reaction to stories told by AI systems.

Another limitation of this work is that the quality of stories written by users may have affected the generated or retrieved stories from ChatGPT and the human-written stories database. This could have downstream effects on the user’s empathy toward the story. Although we did not explore this confound in this paper, comparisons between human-written and AI-written stories were both conditioned on just the user’s story. Our findings indicate that, at large, empathy patterns shift depending on transparency of the author but did not explore personal nuances in the quality of the user’s story. Future works can aim to quantify the quality of written stories and how this might affect empathetic response.

This work focuses on human perceptions of AI story sharing, which can have implications in chatbot design. Such implications are extendable to mental health or social support chatbots that have their own identities or self-disclose their own personal experiences. However, these implications might not apply specifically to chatbots that serve the function of delivering therapy sessions without story sharing. As such, future work should explore the role of transparency regarding machine-like quality or human-like quality in mental health chatbot sessions that are not specific to story sharing.

Finally, there is still a key question to be asked about what role the agent should play in mental health domains, and where empathy fits into this context of human-AI interaction. In traditional patient-therapist relationships, therapeutic alliance, or the working relationship between the two, is a key component and leads to stronger patient outcomes [44]. AI chatbots have been designed to model this type of alliance through verbal empathy or expressing their understanding of the user. The stories presented to the study participants in this study are one way an agent can demonstrate empathy [45]. It is important to note that disclosing personal anecdotes as a form of empathy is different from traditional therapist-client relationships, where the therapists typically shared limited information about themselves [46]. However, there are other supportive relationships or interactions, including companion agents or coaches, that could be mediated by AI technologies. This work paves interesting future directions for how to think about the presentation of model outputs in the context of empathy and personal experiences across a multitude of domains.

### Conclusions

A growing number of companies and research institutions propose using language models and AI chatbots to improve mental well-being or social companionship. Empathy is a core tenet at the center of these chatbot designs, making it crucial to consider the ethical question of how empathy unfolds toward human versus AI narrators, and the role of transparency in this effect. To this end, we conducted 4 crowdsourced studies to assess how empathy differs across human-written versus AI-written stories, varying how stories are selected (generation vs retrieval) and author disclosure (transparency that story was written by an AI author vs no transparency). Although we use current state-of-the-art empathetic retrieval and generation in this work, our findings provide more generalized future insights around human behavior when interacting with AI chatbots. We find that transparency of the author plays an important role in empathy toward an AI story as well as people’s willingness to empathize toward machines. In particular, people generally empathize more with human-written stories but self-report more willingness to empathize with AI, indicating that transparency might play a role in fostering greater trust. This has implications in the design of AI systems intended to empathize *with* or evoke empathy *from* people. Designers of AI applications should consider explainable AI frameworks to make transparent how system content has been generated, as these can affect interaction outcomes. Our work motivates future directions regarding the social, psychological, and ethical implications of nuanced AI system design considerations that can drastically affect the ways in which humans extend empathy to artificial agents in the broader mental health and social support domains.

## Appendix

Multimedia Appendix 1 Full user study task instructions and surveys.

## Abbreviations

AI: artificial intelligence
LLM: large language model
