# Supplementary figures and images for: Empathy Toward Artificial Intelligence Versus Human Experiences and the Role of Transparency in Mental Health and Social Support Chatbot Design: Comparative Study
Source: JMIR Ment Health. 2024 Sep 25;11:e62679. doi: 10.2196/62679 (PMC11464935; doi:10.2196/62679)

# Multimedia Appendix 1

## **Prolific Task Instructions**


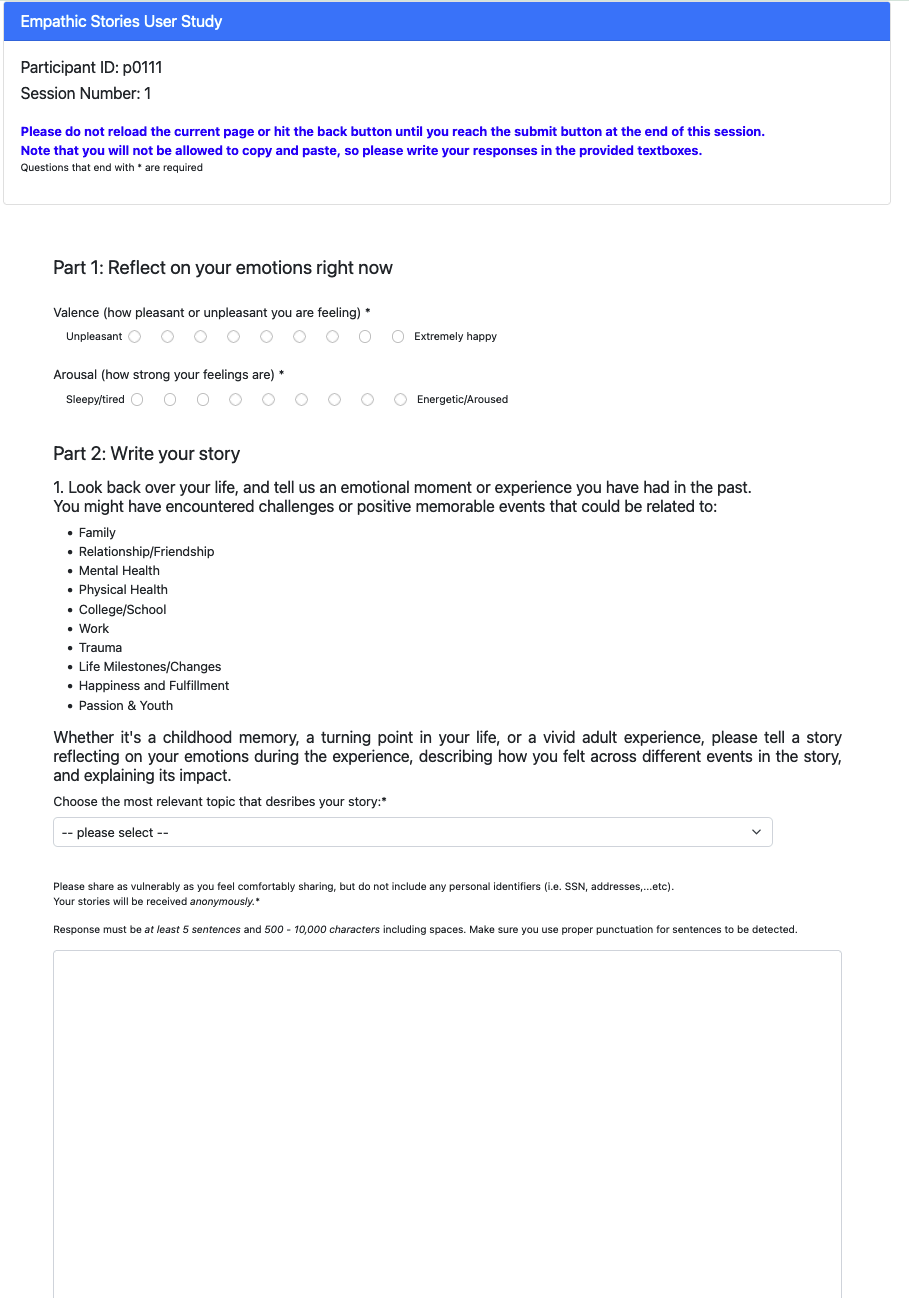


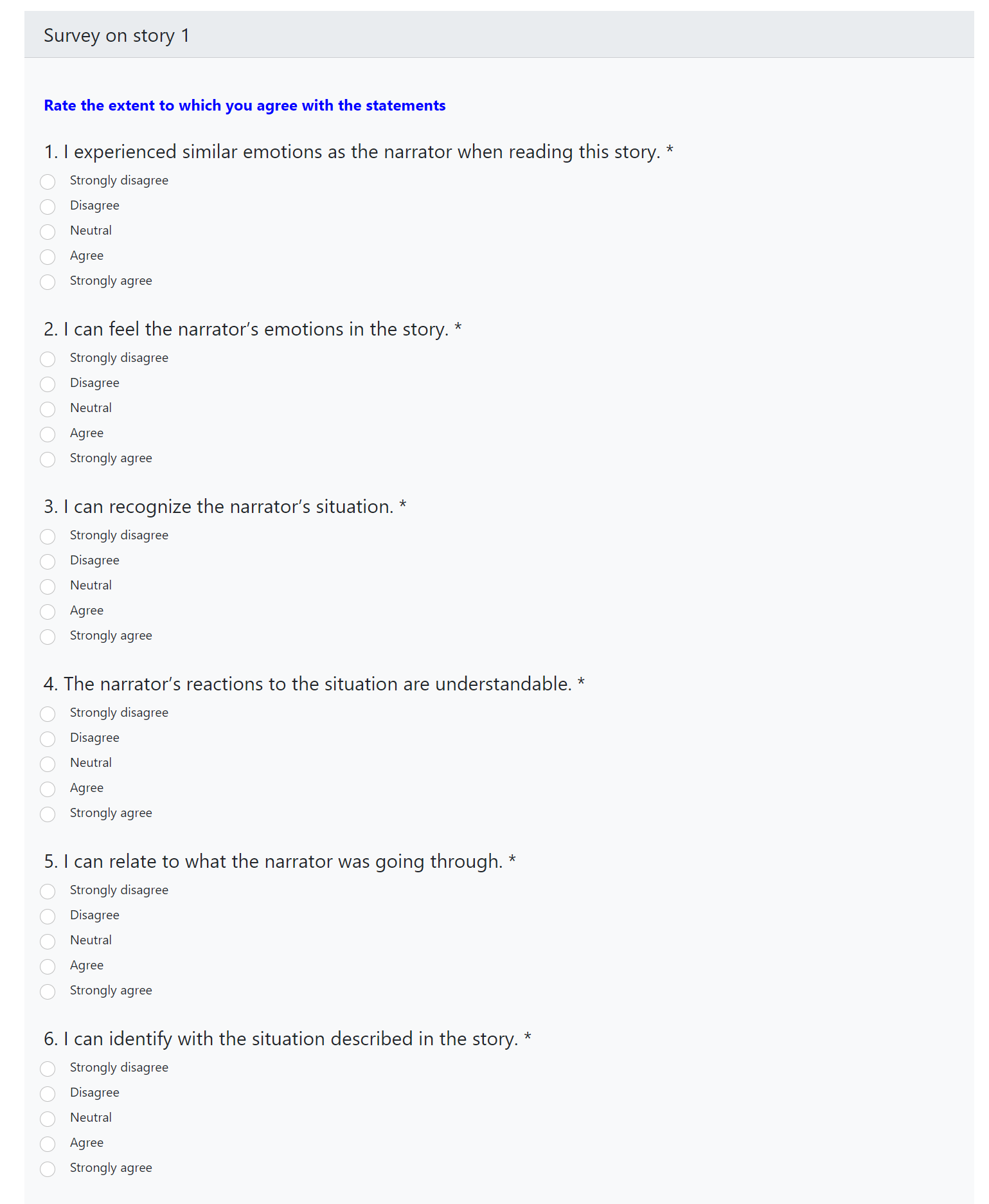

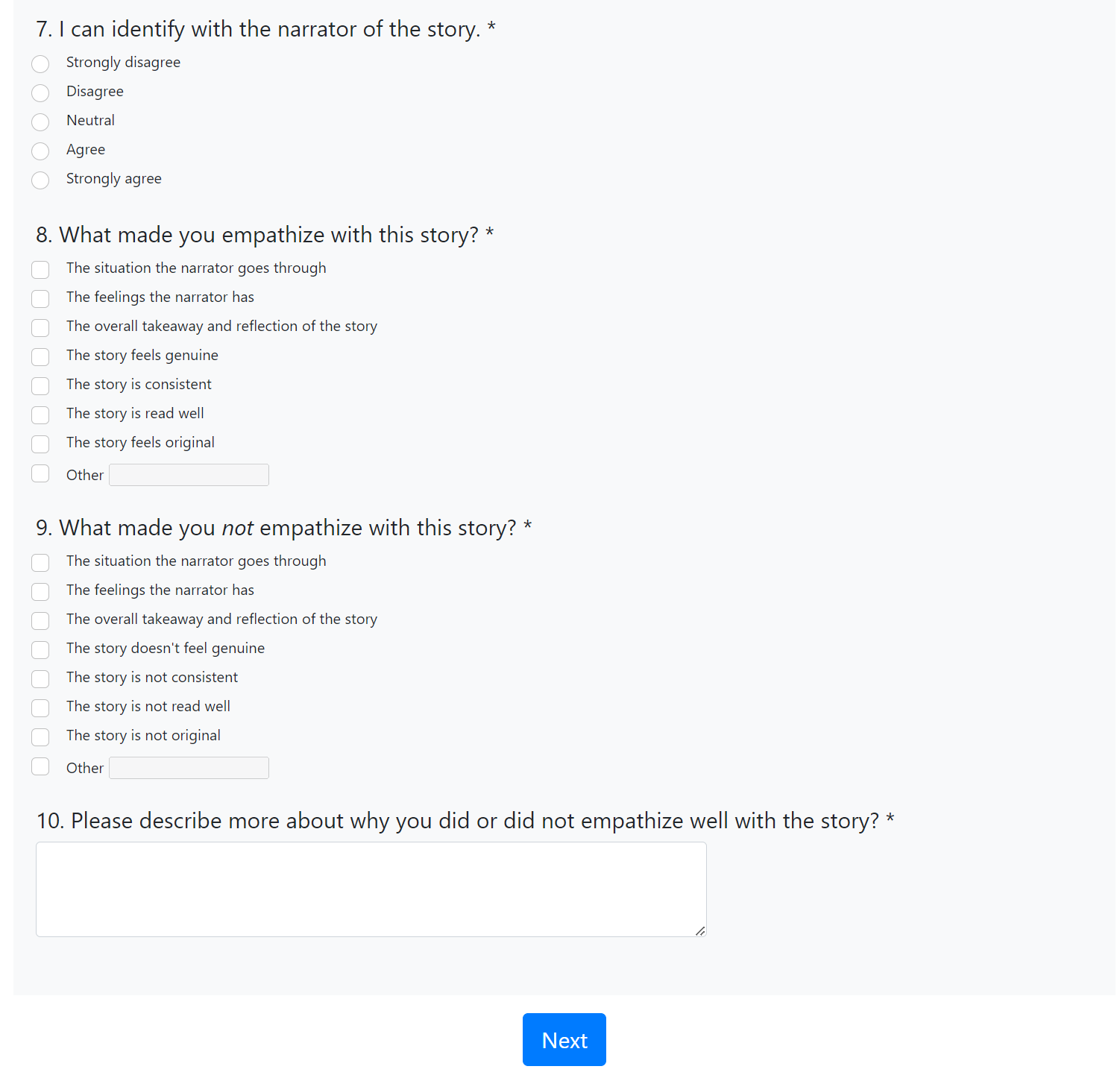

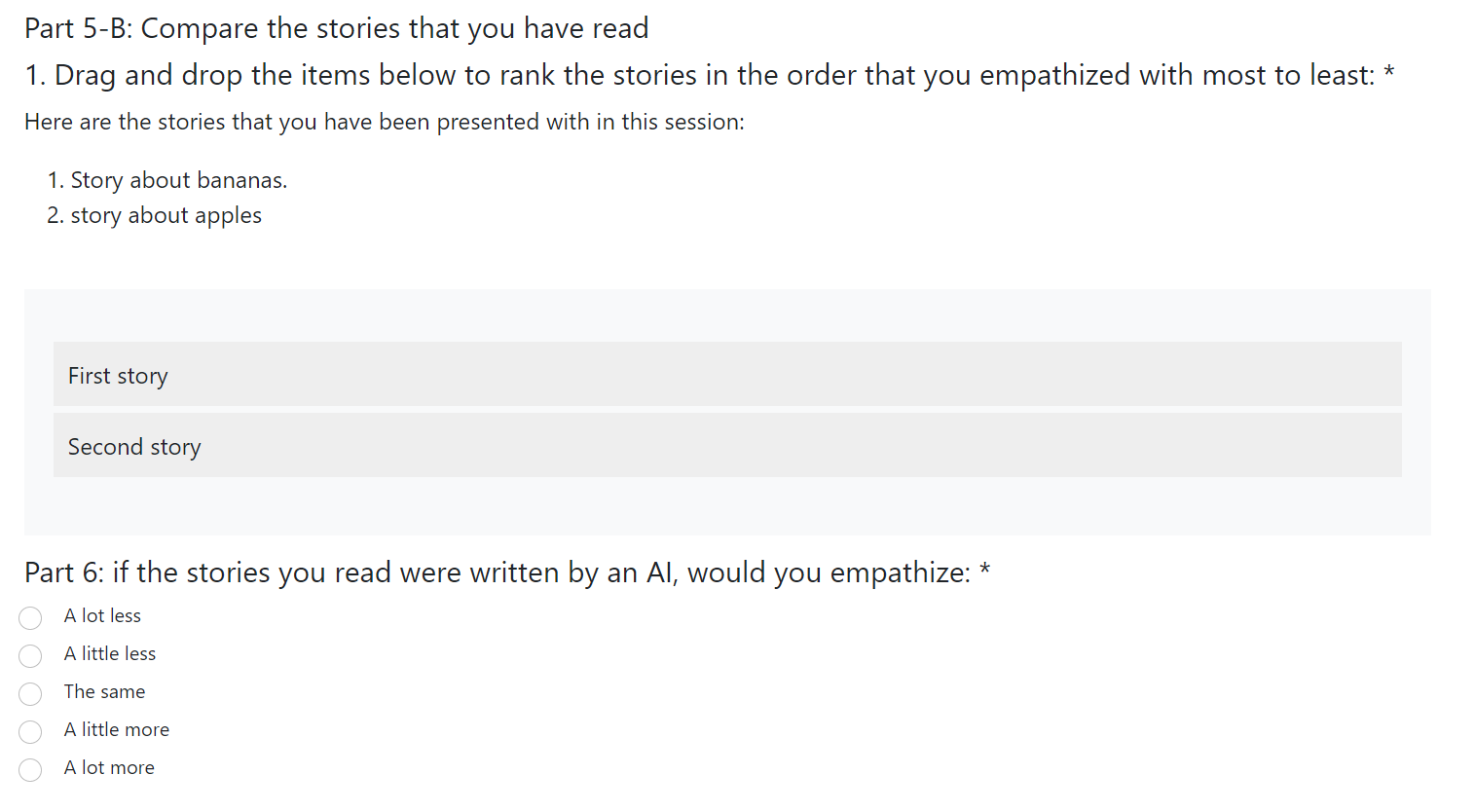

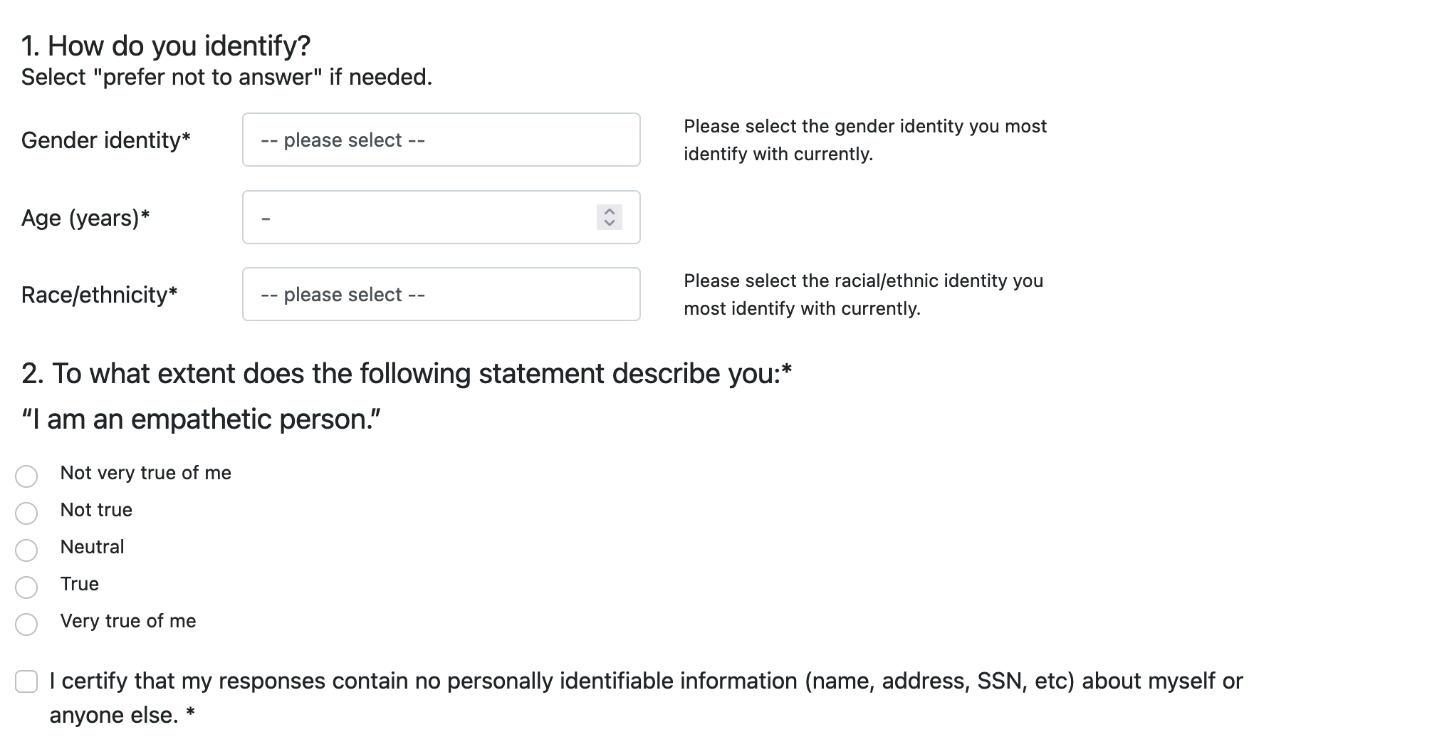

Supplement: Multimedia Appendix 1 [file mental_v11i1e62679_app1.docx]
